# Editorial: Maternal metabolic health: from preconception to postpartum

**DOI:** 10.3389/fnut.2026.1826782

**Published:** 2026-05-14

**Authors:** Javier Diaz-Castro, Jorge Moreno-Fernandez, Maria Luisa Ojeda, Julio J. Ochoa

**Affiliations:** 1 Department of Physiology, Faculty of Pharmacy, University of Granada, Granada, Spain; 2 Institute of Nutrition and Food Technology “José Mataix Verdú”, University of Granada, Granada, Spain; 3 Instituto de Investigación Biosanitaria, Granada, Spain; 4 Department of Physiology, Faculty of Pharmacy, University of Seville, Seville, Spain

**Keywords:** diabetes, early programming, fetal programming, growth, maternal-fetal health, nutrition, post-natal development, preeclampsia

The rising global burden of non-communicable diseases like obesity and hypertension is increasingly linked to early-life programming. This concept suggests that environmental exposures during prenatal and early post-natal periods induce permanent physiological and metabolic changes (1). Because early development involves rapid cellular differentiation and organ maturation, it is a period of high plasticity but also extreme vulnerability. Factors such as maternal nutrition, stress, and toxins can alter developmental trajectories. While these adaptations may aid short-term survival, they often increase disease risk if the post-natal environment differs from fetal expectations (2).

The Developmental Origins of Health and Disease (DOHaD) framework, pioneered by Barker et al. (3), connects low birth weight to adult risks of heart disease and diabetes. In this model, birth weight serves as a proxy for the intrauterine environment—reflecting the combined impact of maternal nutrition, placental function, and endocrine signaling on the programming process (2).

A primary driver of this programming is epigenetic regulation, where environmental factors alter gene expression through DNA methylation and histone modification without changing the DNA sequence (4). Maternal nutrition is one of the most extensively studied determinants of early-life programming. Both maternal undernutrition and overnutrition have been associated with adverse cardiometabolic outcomes in offspring. Evidence has demonstrated that prenatal exposure to severe undernutrition increases the risk of obesity, dyslipidemia, cardiovascular disease, and impaired glucose tolerance in adulthood. The timing of exposure appears to be critical, with early gestational insults exerting particularly pronounced effects on long-term health (5).

In contrast, excessive maternal energy intake, obesity, and gestational diabetes mellitus expose the fetus to hyperglycemia, hyperinsulinemia, and altered lipid availability, promoting increased fetal adiposity and metabolic dysregulation. These changes may predispose offspring to obesity and insulin resistance, thereby contributing to the intergenerational transmission of metabolic disease. Micronutrients also play a crucial role in developmental programming. Nutrients involved in one-carbon metabolism, including folate, vitamin B_12_, choline, and methionine, are essential for DNA methylation and epigenetic regulation. Imbalances in these nutrients during pregnancy may disrupt epigenetic programming and influence long-term disease risk (6).

In this Research Topic there are 21 papers covering several aspects related to maternal-fetal health. A total of eight studies (38% of the total) addressed gestational diabetes. Two investigations examined the relationship between serum mineral concentrations during the second and third trimesters and the risk of gestational diabetes (Hao et al.; Chen, Wu et al.). Another study explored the association between trimethylamine-N-oxide (TMAO), along with its precursor compounds, and gestational diabetes mellitus (GDM) (Chen, Pang et al.). One article reported that the fatty liver index (FLI) is a simple, cost-effective, and easily calculated indicator that may serve as a predictor of GDM risk (Wei et al.), while another study demonstrated that an elevated Zhejiang University (ZJU) index—a novel biomarker for non-alcoholic fatty liver disease (NAFLD) developed in China—in early pregnancy is significantly associated with the development of GDM (Xu, Li et al.). In addition, one study described a postpartum intervention program designed to reduce the long-term risk of diabetes mellitus in women with a history of gestational diabetes (Huang et al.). Another investigation assessed the potential role of myo-inositol supplementation in reducing the risk of GDM and evaluated whether dietary and lifestyle factors modify its effectiveness (Ahmed et al.). Finally, one article examined the combined effects of diabetes mellitus and hypertension on adverse maternal and neonatal outcomes (Jia et al.).

Another study focused specifically on hypertensive disorders by investigating alterations in amino acid metabolism associated with hypertension (Xu, Kong et al.). Among hypertensive disorders of pregnancy, preeclampsia represents one of the most clinically relevant conditions. Evidence suggests a potential association between low maternal vitamin D levels and an increased risk of preeclampsia, particularly when vitamin D deficiency is identified prior to late pregnancy (Zakerinasab et al.). Although vitamin D status has been associated with several adverse outcomes in singleton pregnancies, findings in twin pregnancies remain inconsistent. Dai et al. examined the relationship between maternal vitamin D levels and the progression of twin pregnancies, identifying an association with preeclampsia but not with anthropometric outcomes. Nevertheless, the authors emphasize the importance of monitoring vitamin D status during pregnancy.

With regard to fetal anthropometric outcomes, macrosomia emerges as an important factor associated with adverse neonatal and maternal health outcomes. The study by Luo et al. developed a predictive model aimed at facilitating the early identification of macrosomia risk. Several factors have been associated with macrosomia, including pre-pregnancy body mass index (BMI) and gestational diabetes; however, the interaction between these variables remains incompletely understood. Using a prospectively collected first-trimester pregnancy cohort, Wu et al. examined the association between pre-pregnancy BMI and macrosomia in both GDM and non-GDM populations, concluding that GDM status modifies the relationship between pre-pregnancy BMI and macrosomia risk. Pre-pregnancy BMI has also been investigated in relation to offspring neurobehavioral development. Sun et al. reported that maternal pre-pregnancy BMI should be considered an independent risk factor for neurobehavioral outcomes in offspring, with effects that may differ according to sex.

This Research Topic also includes a study examining the relationship between vitamin C intake and fertility, and how this association may be influenced by maternal depression and body mass index (Zheng, Ren et al.). Another study investigated the association between mitochondrial DNA copy number and different patterns of intrauterine growth (https://doi.org/10.3389/fped.2025.1569702Chen, Li et al.). Zheng, Zhang et al. applied machine learning techniques to develop a predictive model for the risk of premature rupture of membranes, examining its association with inflammatory and nutritional indices. Additional research in the Research Topic explores the influence of dietary habits (Ma et al.) and physical activity (Wang et al.) on pregnancy progression and maternal–fetal health outcomes. Finally, one study evaluated the effectiveness of a personalized intervention to improve calcium intake during pregnancy (Heidari et al.), while another highlighted the need for standardized diagnostic and therapeutic protocols for rectal ectopic pregnancy (Zhang et al.).

Emerging evidence suggests that the effects of early-life programming may extend beyond a single generation. Maternal metabolic health influences not only fetal development but may also affect the germline, thereby contributing to intergenerational transmission of disease risk. Animal studies have demonstrated transgenerational inheritance of epigenetic modifications induced by nutritional and environmental exposures, hile human studies suggest similar patterns, although causal pathways remain difficult to establish. The recognition that disease risk may be established early in life has important implications for prevention. Interventions aimed at optimizing maternal nutrition, metabolic health, and prenatal care represent a critical opportunity to reduce the burden of chronic disease at the population level. The DOHaD framework supports a life-course approach to health, emphasizing prevention strategies that begin before conception and extend through pregnancy and early childhood (7).

## Conclusion

Environmental exposures during sensitive windows of prenatal and early post-natal development—particularly maternal nutrition, metabolic status, stress, and placental function—can induce lasting structural, physiological, and epigenetic changes that influence susceptibility to non-communicable diseases across the life course. The DOHaD framework has provided a robust conceptual model linking intrauterine conditions to adult cardiometabolic outcomes, highlighting that disease risk may be established well before the onset of clinical symptoms. From a public health perspective, these findings highlight the importance of adopting a life-course approach to disease prevention, with particular emphasis on optimizing maternal health before and during pregnancy. Interventions targeting maternal nutrition, metabolic control, and prenatal care represent critical opportunities to mitigate the rising burden of chronic non-communicable diseases. Continued research aimed at elucidating precise mechanisms, identifying critical windows of susceptibility, and developing effective early-life interventions will be essential for translating the principles of developmental programming into strategies that improve population health outcomes.
